# Selectivity filters and cysteine-rich extracellular loops in voltage-gated sodium, calcium, and NALCN channels

**DOI:** 10.3389/fphys.2015.00153

**Published:** 2015-05-19

**Authors:** Robert F. Stephens, W. Guan, Boris S. Zhorov, J. David Spafford

**Affiliations:** ^1^Department of Biology, University of WaterlooWaterloo, ON, Canada; ^2^Department of Biochemistry and Biomedical Sciences, McMaster UniversityHamilton, ON, Canada; ^3^Sechenov Institute of Evolutionary Physiology and Biochemistry, Russian Academy of SciencesSt. Petersburg, Russia

**Keywords:** calcium channels, sodium channels, NALCN, ion selectivity, ion channel evolution, ion channels, *Lymnaea stagnalis*, ion pore

## Abstract

How nature discriminates sodium from calcium ions in eukaryotic channels has been difficult to resolve because they contain four homologous, but markedly different repeat domains. We glean clues from analyzing the changing pore region in sodium, calcium and NALCN channels, from single-cell eukaryotes to mammals. Alternative splicing in invertebrate homologs provides insights into different structural features underlying calcium and sodium selectivity. NALCN generates alternative ion selectivity with splicing that changes the high field strength (HFS) site at the narrowest level of the hourglass shaped pore where the selectivity filter is located. Alternative splicing creates NALCN isoforms, in which the HFS site has a ring of glutamates contributed by all four repeat domains (EEEE), or three glutamates and a lysine residue in the third (EEKE) or second (EKEE) position. Alternative splicing provides sodium and/or calcium selectivity in T-type channels with extracellular loops between S5 and P-helices (S5P) of different lengths that contain three or five cysteines. All eukaryotic channels have a set of eight core cysteines in extracellular regions, but the T-type channels have an infusion of 4–12 extra cysteines in extracellular regions. The pattern of conservation suggests a possible pairing of long loops in Domains I and III, which are bridged with core cysteines in NALCN, Cav, and Nav channels, and pairing of shorter loops in Domains II and IV in T-type channel through disulfide bonds involving T-type specific cysteines. Extracellular turrets of increasing lengths in potassium channels (Kir2.2, hERG, and K2P1) contribute to a changing landscape above the pore selectivity filter that can limit drug access and serve as an ion pre-filter before ions reach the pore selectivity filter below. Pairing of extended loops likely contributes to the large extracellular appendage as seen in single particle electron cryo-microscopy images of the eel Na_v_1 channel.

## Complexities in resolving the 4x6TM family of cation channels

The mechanism of sodium and calcium selectivity within the superfamily of eukaryotic 4x6TM ion channels, which includes the voltage-gated sodium channels (Na_v_1, Na_v_2), voltage-gated calcium channels (Ca_v_1, Ca_v_2, Ca_v_3) and NALCN, is not well understood. Invertebrate NALCN and T-type channels demonstrate mixed sodium and calcium selectivity due to alternative splicing involving (i) the HFS site at the pore selectivity filter and (ii) cysteine-rich extracellular turrets (Senatore et al., 2013, 2014a,b). We use available phylogenetic data on 4x6TM channels to examine different molecular determinants that underline sodium and calcium selectivity in these channels. NALCN and Na_v_1 sodium channels have a HFS site where sodium selectivity is generated by a lysine residue in the second or third repeat domain. We find a core of eight conserved cysteines in the long extracellular loops of all 4x6TM channels, and additional cysteines in the loops of Ca_v_3 T-type channels and many of the vertebrate Na_v_1 channels. The DI-DIII and DII-DIV pairing of extracellular loops creates an appendage above the pore selectivity filter. Such appendages are seen in the single nanoparticle cryo-electron microscopy images of Na_v_1 sodium channels and in the X-ray structures of K2P1 channels, where the disulfide bond-bridged extended extracellular turrets form an apex ~35 angstroms above the membrane. In K2P1 channels this appendage affects ion and drug access to the pore. Similar appendages contribute to sodium selectivity engendered in invertebrate T-type channels with alternative cysteine-rich extracellular turrets. Other resources on related topics cover structure of the prokaryotic sodium channels (Charalambous and Wallace, 2011; Payandeh et al., 2011; Catterall, 2014; Scheuer, 2014; Payandeh and Minor, 2015), homology modeling of eukaryotic sodium channels (Tikhonov and Zhorov, 2012; Korkosh et al., 2014) and a proposed evolution of sodium and calcium channels (Liebeskind et al., 2011; Cai, 2012; Zakon, 2012; Moran et al., 2015).

**Figure 1:**
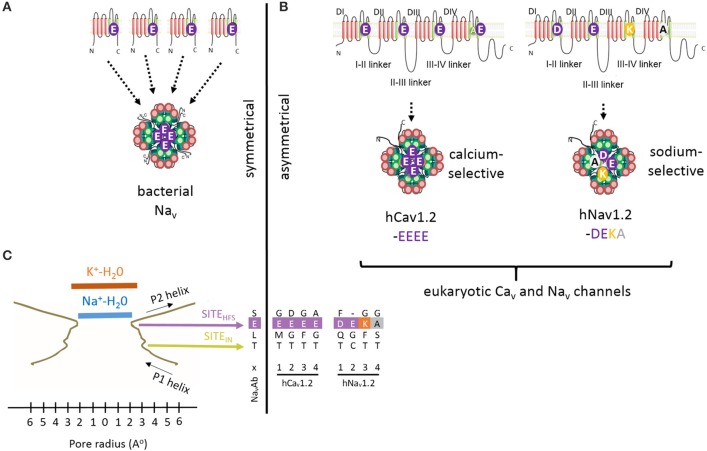
**High Field Strength (HFS) site EEEE formed by selectivity filter glutamates (E177) in the homotetrameric bacterial sodium channel NavAb is equivalent to the EEEE site in of Ca_v_1 and Ca_v_2 calcium channels and the DEKA site of Na_v_1 sodium channels, which are contributed by the four homologous repeat domains. (A)** Residues to the EEEE HFS site in bacterial sodium channels are contributed by four identical gene products. **(B)** Residues to asymmetric selectivity filters in eukaryotic calcium and sodium channels are contributed by four homologous repeat domains. **(C)** Structural contours of the NavAb selectivity filter and positions of corresponding residues of bacterial NavAb and human Ca_v_1.2 and Na_v_1.2. The narrowest pore level is the selectivity filter HFS site, which can accommodate a hydrated Na^+^ ion, but not a hydrated K^+^ ion. Panel**(C)** is adapted from Payandeh et al. (2011), with permission.

“4x6TM” refers to the shared architecture in the superfamily of cation channels that contain four homologous repeat domains with six transmembrane segments in each domain (Figure 1). 4x6TM channels arose from two rounds of duplication in a 1x6TM channel ancestor. The evidence for this evolutionary history is in the kinship of the domains, where the pairs of Domains I and III, and Domains II and IV more closely resemble each other than other domains in these channels (Strong et al., 1993). Examples of the 1x6TM genes are bacterial sodium channels (e.g., NavAb) (Payandeh et al., 2011; Zhang et al., 2012) (Figure 1A) and voltage-gated potassium channels (Gutman et al., 2005). Four repeat domains in the 4x6TM channels or four subunits in the 1x6TM channels form an assembly that surrounds the aqueous pore through which ions pass (Figure 1). In the 1x6TM channels, four subunits can form homo-tetramers derived from identical gene products, or may co-assemble with subunits from different gene products as a hetero-tetramer, as in Shaker type voltage-gated potassium channels (Li et al., 1992) (Figure 1A). A consequence of four repeat domains linked in a full-length 4x6TM channel gene is the absence of the diversity that is observed in hetero-tetrameric voltage-gated potassium channels. What is gained in the 4x6TM genes is a sequential asymmetry of their repeat domains, where each domain is substantially diverged from other domains, and can make a unique contribution to the voltage-gating, ion selectivity and interactions with the cytoplasmic or extracellular environments. The four repeat domains are connected by different cytoplasmic linkers (I-II, II-III, and III-IV), as opposed to four pairs of cytoplasmic N- and C-termini in K channels (Figure 1B). These linkers possess regulatory sites that interact with proteins, such as auxiliary channel subunits. The linkers are diversified for unique cellular conditions. Examples of such conditions include the axonal specific environment associated with Na_v_1 sodium channels in nodes of Ranvier of myelinated neurons (Leterrier et al., 2011), or the network of proteins appearing to associate with Ca_v_2 channels in presynaptic terminals (Spafford and Zamponi, 2003). While K channels also have unique protein interacting domains, these are mostly limited to the N- and C-termini, which are fourfold repeated in each K channel (Jan and Jan, 2012).

It is challenging to study 4x6TM channels by site-directed mutagenesis or by generating chimeric channels where the inter-dependence of individual repeat domains is not understood. 4x6TM channel structures are also difficult to resolve by X-ray crystallography because of their large size (~2.1 to ~2.9 × 10^3^ amino acids), large extra- and intra-cellular loops, as well as N- and C-terminal ends. Single nanoparticle cryo-electron microscopy and advanced direct detection cameras may provide greater insights into the structure of 4x6TM channels (Liao et al., 2013, 2014). Hypotheses on the structure of ion pores in 4x6TM channels in this review are proposed using the following sources: (i) comparison of known X-ray structures in 1x6TM channels, (ii) comparative analyses of sequences and physiological characteristics of Na_v_1, Na_v_2, Ca_v_1, Ca_v_2, Ca_v_3 and NALCN channels, (iii) analysis of the full phylogenetic spectrum of channels starting from early eukaryotes where 4x6TM channels first appear.

## The modularity of 4x6TM channel domains

**Figure 2:**
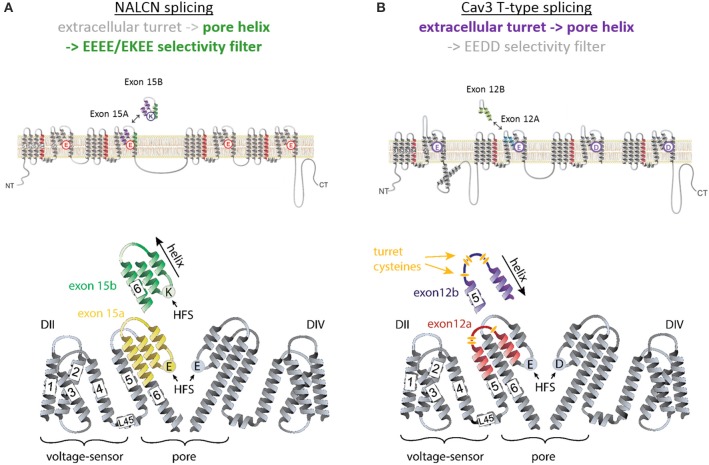
**Invertebrate NALCN and T-type channels splice in the pore module (Domain II) to generate calcium- or sodium-selective channels**. Only repeat Domains II and IV are shown for clarity. Each repeat domain contains a voltage-sensing module (S1 to S4) and segments S5-P-S6 that contribute a quarter to the pore module. **(A)** Exons 15a or 15b code for alternative HFS sites generating calcium-selective (EEEE) or sodium-selective (EKEE) pores (Senatore et al., 2013). **(B)** Expression of exon 12a or 12b upstream of the HFS site in the Ca_v_3 T-Type channels generates alternative ion selectivity in the of the same invertebrate species (giant pond snail *Lymnaea stagnalis*). The exons code for extracellular loops with three or five cysteines that engender the invertebrate T-type channels with greater sodium selectivity or less sodium selectivity, respectively (Senatore et al., 2014a).

Each of four homologous domains in a 4x6TM channel and the single domain in a 1x6TM channel consists of a voltage-sensing module (involving transmembrane helices S1 to S4) and a quarter of the pore module (involving transmembrane segments S5 and S6 and membrane re-entrant P-loop between S5 and S6) (Figure 2). Some voltage sensing and pore-forming modules exist as natively expressed stand-alone proteins. Examples include a proton-gated channel Hv1 that lacks the pore module (Ramsey et al., 2006; Sasaki et al., 2006) and the pH-activated bacterial KcsA potassium channel that lacks voltage-sensing modules (Doyle et al., 1998). The voltage sensor and pore modules exist as natively expressed proteins on their own, such as the voltage-sensor containing phosphatase Ci-VSP from the sea squirt *Ciona intestinalis* (Murata et al., 2005), and the bacterial KcsA potassium channel, a pH-activated protein that has only the pore module (Doyle et al., 1998). Truncated subunits of bacterial sodium channels can form *in vitro* functional tetramers without the voltage-sensor domains (McCusker et al., 2011; Tsai et al., 2013; Shaya et al., 2014), illustrating the semi-autonomous nature of the voltage-sensing and pore modules in voltage-gated channels. However, it is typical in the 4X6TM channels for the voltage-sensing module and pore module to appear together.

The voltage sensing helix S4 contains several positively charged residues located on one face of the helix. Upon membrane depolarization S4 moves in the extracellular direction along helices S2 and S3 that contain negatively charged residues (Catterall, 2010) (Figure 2). Amphipathic S4-S5 linkers, which connect the voltage sensing and pore modules, allow movements in the voltage sensors to induce movements of the S5 helices in the pore module. As a result, the innermost S6 segments, which line the aqueous pore and form a helical bundle, can widen or narrow to allow or occlude ion permeation (Oelstrom et al., 2014).

## P-loop residues that contribute to the selectivity filter

Each subunit or repeat domain of a P-loop channel contains transmembrane helices S5 and S6 that are connected by the membrane-re-entrant P-loop. Each P-loop contains an extracellular turret-like linker (S5P) between S5 and a membrane-descending P-helix, an ascending limb, and an extracellular linker to S6. Four ascending limbs contain residues that contribute to the selectivity filter and line the outer pore (Figure 2). At the narrowest level of the pore within the P-loop domain, the four ascending limbs contain residues that determine the K^+^, Ca^2+^, Na^+^, or mixed ion selectivity, and divide the ion permeation pathway into an outer pore above and the inner pore below the selective filter.

### Potassium ion selectivity

**Figure 3:**
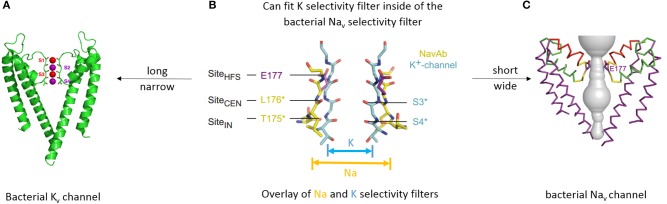
**Different structures underlying different mechanisms of selectivity in potassium and sodium channels. (A)** X-ray structure of bacterial KcsA potassium channel **(A)** (Doyle et al., 1998). Only two subunits are shown for clarity. Backbone carbonyls in the selectivity filter are red. Potassium ions (purple spheres) occupy two of the four sites (s2 and s4). Water molecules (red spheres) are shown in positions s1 and s3. **(B)** Overlay of the selectivity filter regions in KcsA and NavAb. NavAb (yellow carbons) has a shorter and wider pore in the selectivity-filter region. **(C)** X-ray structure of bacterial sodium channel NavAb (Payandeh et al., 2011). P2 helices are red and high field strength site glutamate residues (E177) are purple. **(B,C)** are reproduced from Payandeh et al. (2011), with permission.

The potassium ion selectivity in K channels is determined by backbone carbonyl oxygens (Doyle et al., 1998) from the signature sequence residues (TVGYG), which mimic the hydration shell oxygen atoms that surround potassium ions in solution (Figure 3A). There are four potassium binding sites in the selectivity filter (s1-s4), which in the current-permeating channel can be occupied by two water molecules and two potassium ions (s1/s3 or s2/s4 potassium occupancy) (Figure 3A).

### Sodium ion selectivity

The general folding for the 4x6TM channels likely resembles that seen in the X-ray structures of the 1x6TM bacterial sodium channels NavAb (Figures 3A,C) (Payandeh et al., 2011), NavRh (Zhang et al., 2012), and NavMs (McCusker et al., 2012). The selectivity filter of bacterial Nav channels contains a ring of four glutamates (EEEE), one glutamate from each subunit (Figure 3C). The EEEE ring forms the narrowest level of the open pore (Figure 1C) with the flexible carboxylate side chains facing toward the pore axis. The conformational flexibility of the carboxylate side chains stabilizes multiple ionic occupancy states, helping sodium ions to pass through the selectivity filter via the knock-on mechanism (Chakrabarti et al., 2013).

## Selectivity filter residues in eukaryotic sodium and calcium channels

**Figure 4:**
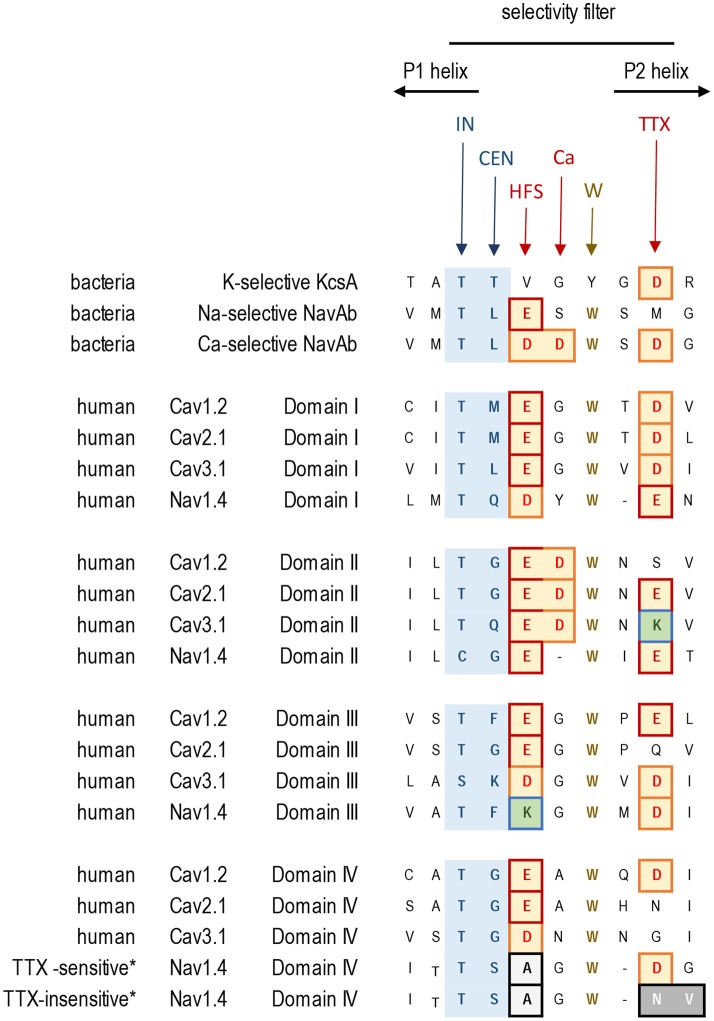
**Alignment of sequences contributing to the selectivity filters in K^+^, Ca^2+^, and Na^+^ channels**. Residues contributing to the central (CEN) and inner (IN) sites at the selectivity filter region are highlighted blue. Negatively charged residues contributing to the HFS sites and to the rings of outer carboxylates are red/brown. The aspartate residue in Domain II, which is next to the HFS site, is conserved in Ca_v_1, Ca_v_2, and Ca_v_3 channels. Exceptionally conserved tryptophans form inter-repeat hydrogen bonds that stabilize the P-loop folding. Note the difference in Domain IV of the TTX-sensitive (hNa_v_1.4) and TTX-low-sensitive channels from garter snake (*Thamnophis sirtalis*) that adapted to feed on TTX-ladened newts by neutralizing a negative charge in the TTX site.

**Figure 5:**
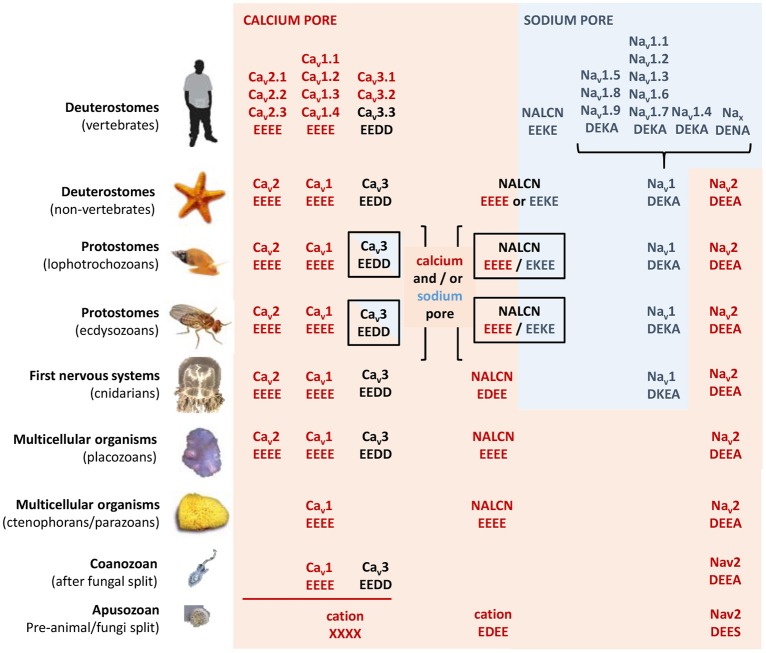
**Evolution of calcium and sodium HFS sites in 4x6TM channels**. Most basal metazoan species have only calcium-selective pores: Ca_v_1 (EEEE), Ca_v_2 (EEEE), Na_v_2 (DEEA), and NALCN (EEEE). Sodium selective HFS sites contain a lysine residue (the DEKA ring) in most animal groups. Exceptions are the simplest animals with sodium-dependent action potentials (cnidarians) that have a DKEA HFS site (Anderson et al., 1993; Spafford et al., 1998). Dual sodium and calcium selectivity in NALCN is due to alternative splicing at HFS site with exon 15 or exon 31 (Senatore et al., 2013). T-Type calcium channels with mixed sodium and calcium selectivities have a shorter extracellular loop with three cysteines or a longer loop with five cysteines in Domain II coded by exons 12a and 12b, respectively (Senatore et al., 2014a). Vertebrates have sodium-selective channels. They lack calcium-selective Na_v_2 channels and the NALCN splice isoform with the calcium selectivity filter (EEEE). Vertebrates also lack sodium-selective T-Type channels as found in the invertebrate heart.

The HFS site at the selectivity filter of eukaryotic Ca_v_1 and Ca_v_2 calcium channel is formed by a ring of negatively charged glutamates (EEEE), which resembles the EEEE ring of bacterial NavAb channels (Figures 4, 5). Ca_v_3 (T-type) channels have a ring of four acidic residues (EEDD) with the third and fourth domains providing aspartates rather than glutamates (Figures 4, 5).

In eukaryotic Na_v_1 sodium channels, the selectivity filter includes a protonatable lysine (K) from the third repeat and a neutral alanine (A) from the fourth repeat (the DEKA ring). Cnidarians, which possess the simplest body plan to include a nervous system and the most basal organisms with Na_v_1 channels are the exception. Instead of the to the DEKA ring cnidarian selectivity filters have lysine and glutamate provided by the second and third repeats, respectively (the DKEA ring) (Figure 5). The critical importance of the selectivity-filter residues was established in experiments where the DKEA ring in the Na_v_1.2 channel was replaced by the EEEE ring to create calcium-selective channels (Heinemann et al., 1992; Schlief et al., 1996).

The single HFS site is a key determinant providing selectivity for calcium- and sodium-selective channels. The EEEE ring is present in all Ca_v_1/Ca_v_2 channels and EEDD is present in all Ca_v_3 channels found in every known animal species to date, down to the single cell coanoflagellates in which the Ca_v_1 and Ca_v_3 calcium channels first appeared (Figure 5). All metazoans that have highly sodium selective Na_v_1 channels, from cnidarians to humans, have Na_v_1 channels with the DEKA, DEKG, or DKEA HFS sites (Figure 5). The first sodium-selective Na_v_1 channels (Spafford et al., 1998) and neuronal currents (Spafford et al., 1996) may have appeared in extant cnidarians, which are the simplest organisms with a nervous system. Na_v_1 channels likely evolved as an offshoot of the more primordial Na_v_2 channel, which is a calcium-selective channel in non-vertebrate species such as insects (Zhou et al., 2004) and cnidarians (Gur Barzilai et al., 2012), containing the DEEA or DEES rings in the HFS (Figure 5). Notably, these Na_v_2 channels are lacking the third-repeat lysine, which is present in the Na_v_1 channels.

## Channels with P1 and P2 helices in P-loops

X-ray structures of bacterial sodium channels show a descending helix (P1) and an ascending helix (P2), with the selectivity-filter region between P1 and P2 (Figures 1C, 3C, 4). The ascending P2 helix is absent in potassium channels (Figure 2A), but is likely present in eukaryotic 4X6TM sodium and calcium channels (Tikhonov and Zhorov, 2012). The outer vestibule is formed by P2 helices, which contain additional negatively charged residues positioned to attract cations to the pore. In sodium channels these residues are targeted by conotoxins, (see Korkosh et al., 2014) and references therein. The cluster of negatively charged residues likely forms binding sites for incoming cations above the HFS site. Indeed, bacterial NavAb channel with three engineered aspartates in the HFS site and P2 helix (replacement of TL***ES***WS***M*** by TL***DD***WS***D***) demonstrates calcium selectivity (Yue et al., 2002; Tang et al., 2014) (Figure 4).

In the same position where the second aspartate substitution (TLD***D***WSD) generates the calcium-selective bacterial sodium channel, the aspartate in the second repeat is conserved universally in all eukaryotic calcium channels, including Ca_v_1, Ca_v_2 and Ca_v_3 channels from single cell coanoflagellates to mammalian channels (Tikhonov and Zhorov, 2011; Payandeh and Minor, 2015). This aspartate, which is next to the HFS site glutamate in Domain II, (e.g., TGE***D***WNS in Ca_v_1.2, see “Ca” site in Figure 4), is likely required for calcium ion selectivity.

Negatively charged glutamate or aspartate residues in the P2 helix, three or four positions downstream from the HFS site form an outer ring that is common in 4x6TM calcium and sodium channels (see “TTX” site, Figure 4). These positions correspond to the third aspartate residue in the TLDDWS***D*** motif that contributes to the engineered calcium selectivity in the NavAb channel construct. Other negative charges, positioned more distantly and above the HSF in the outer vestibule, have more modest effects on ion selectivity (Chiamvimonvat et al., 1996; Favre et al., 1996; Schlief et al., 1996) and they are not conserved among all 4x6TM channels or within different sodium and calcium channel subtypes. The rather poor conservation of negative charges in the outer vestibule contrasts with the invariant EEEE HFS site and the adjoining aspartate just above the HFS site glutamate in Domain II, which are likely key determinants for calcium selectivity (Tikhonov and Zhorov, 2011; Payandeh and Minor, 2015).

## Tetrodotoxin resistance and P2 helix residues

Selective pressures due to the presence of pore-blocking toxins have led to adaptive resistance in certain organisms, involving substitutions in the sodium channel residues three and four positions distant from the HFS site. Alterations in sodium channel pores can be used to escape the influences of tetrodotoxin (TTX). This highly potent neurotoxin is generated by symbiotic bacteria that penetrate animal tissues of TTX-bearing animals. The latter include many invertebrate species (snails, crabs) as well as vertebrates such as tetraodontiform fish (e.g., pufferfish) or newts. Highly TTX sensitive vertebrate nerve and skeletal muscle-specific sodium channels possess an outer ring of negatively charged residues, EEDD, perched above the DEKA HFS site (e.g., the TSAGWD sequence in Domain IV of the Na_v_1.4 channel, see “TTX” site, Figure 4). TTX resistance can be generated by neutralizing this outer ring of negatively charged residues (Terlau et al., 1991). Invertebrates possess only one Na_v_1 channel gene, and almost complete TTX resistance is generated in many invertebrate species (e.g., jellyfish, some flatworms, pulmonate snails, *Varroa* mite and sea squirts) due to the lack of negative charges in the outer ring (Du et al., 2009). Particular populations of common garter snake that feed on TTX-ladened newts have similarly altered Na_v_1 channels with a neutralized outer ring aspartate in Domain IV, most prevalently in skeletal muscle Na_v_1.4 sodium channels (Figure 4) (Feldman et al., 2012). This is an example of rapid intra-species evolution of sodium channels, since common garter snake populations that do not feed on TTX-ladened newts in their locale, possess unaltered, TTX-sensitive Na_v_1.4 sodium channels (Feldman et al., 2012) (Figure 4). There are also Domain IV mutations in Na_v_1.4 sodium channels of pufferfish and other species (Jost et al., 2008), which prevent self-poisoning by TTX generated in their own tissues.

## Mixed calcium- and sodium-selective HFS sites in NALCN ion channels

**Figure 6:**
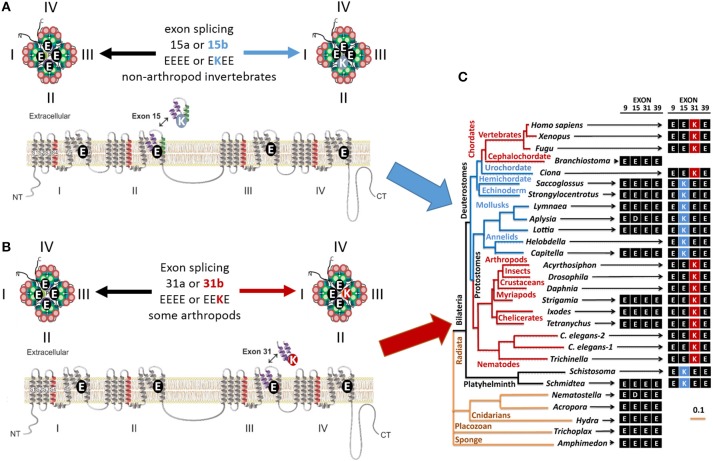
**Gene splicing in alternative calcium and sodium HFS sites in NALCN channels**. In simple multicellular animals (sponges and placozoans) whose bodies lack organs, NALCN homologs have the selectivity filters like in calcium-selective pores (EDEE or EEEE). **(A)** Duplication of Exon 15 that provides Domain II with alternative glutamate (E) or lysine (K) residue for the HFS site creates alternative calcium-selective (EEEE) or sodium-selective (EKEE) pores in different organisms. These include platyhelminthes, the planarian (*Schmidtea*), protostomes of the lophotrochozoan lineage (mollusks and annelids), and non-chordate deuterostomes (echinoderms and hemichordates). **(B)** Duplication of exon 31 in the ecdysozoan lineage provide in Domain III with alternative glutamate (E) or lysine (K) residue for the HFS site. Splicing in exon 31 generates alternative calcium-selective (EEEE) and sodium-selective (EEKE) HFS sites retained in myriapods (includes centipedes, millipedes) and chelicerates (includes Arachnids like mites and ticks). **(C)** The NALCN gene tree illustrating the retention of the calcium-selective HFS site in the only NALCN isoform in the simplest metazoans (sponge, placozoan, and cnidarians) and the cephalochordate (*Branchiostomia*, amphioxus). Animals retaining only the sodium-selective HFS site include nematodes, some arthropods and vertebrates. Adapted from Senatore et al. (2013).

NALCN ion channels provide a different example of adaptive evolution, and it involves modifying the HFS site, as a means to generate alternative ion selectivities (Senatore et al., 2013). NALCN is a separate lineage of 4x6TM channels with the ion selectivity in different species that can resemble either or both calcium-selective Ca_v_1 and Ca_v_2 channels, which have the EEEE HFS site, and the sodium selective Na_v_1 channels, which have the DEKA, DEKG, or DKEA HFS sites (Senatore et al., 2013) (Figures 2, 6). NALCN channels possess a HFS site of a calcium channel (EEEE) in basal multicellular organisms (sponge, placozoan and cnidarians) and non-vertebrate chordates (the cephalochordates) (Senatore et al., 2013) (Figure 6). However, in other animals, NALCN channels resemble sodium channels with a lysine in Domain II or III of the HFS site, respectively. Examples include the EKEE ring in *schistosome* flatworms and *Helobdella* leech and the EEKE ring in non-myriapod arthropods and vertebrates (Senatore et al., 2013) (Figure 6). A separate group of non-vertebrates retain dual calcium and sodium selectivity at the HFS site, through mutually exclusive splicing, and these evolved completely independently in at least two different animal lineages. Almost all lophotrochozoan invertebrates (mollusks, annelids) and non-vertebrate deuterostomes (echinoderms, hemichordates) possess two forms of exon 15 in their expressible NALCN transcripts, generating alternative calcium HFS site (EEEE) with exon 15a or a sodium HFS site (EKEE) with exon 15b (Senatore et al., 2013) (Figure 6). Non-arthropod ecdysozoan protostomes (myriapods, chelicerates) possess two forms of exon 31 generating an alternative calcium HFS site (EEEE) with exon 31a or a sodium HFS site (EEKE) with exon 31b (Senatore et al., 2013) (Figure 6).

There is compelling evidence from nematode (*C. elegans*), fruit fly (*Drosophila*) and mammals that NALCN channels contribute to pacemaker currents that lead to rhythmic activity such as locomotion (Gao et al., 2015), circadian clock rhythms (Lear et al., 2013), and breathing (Lu et al., 2007). Why animals have a calcium-selective or sodium-selective form of NALCN, or both calcium and sodium selectivities in their NALCN channels through alternative splicing is not yet understood. Invertebrate (Senatore et al., 2013) and mammalian (Lu et al., 2007; Swayne et al., 2009; Chong et al., 2015) NALCN channels express *in vitro*, but it has been challenging to separate NALCN currents (Lu et al., 2007) from those generated from leaky patch seals (Boone et al., 2014).

## Dual sodium and calcium selectivities in Ca_v_3 T-type channels

**Figure 7:**
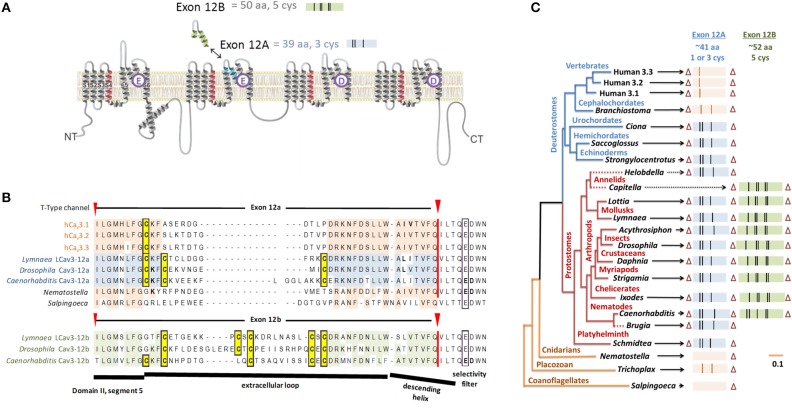
**Gene splicing producing different cysteine-rich extracellular loops in T-type channels with alternative calcium and sodium selectivities. (A)** In snails, shorter exon 12a with three cysteines creates highly sodium selective T-type channels and the longer exon 12b with five cysteines generates more calcium selective T-type channels. **(B)** Alignment of representative species containing exon 12a/12b with conserved cysteine residues (highlighted yellow). **(C)** The gene tree of exon 12a/12b of T-type channels. Exon lengths are scaled with cysteine residues indicated as vertical bars. Most protostome invertebrates contain a shorter exon 12a with a three cysteines (blue) and an alternative exon 12b with five cysteines (green). All vertebrate T-type channels have exon 12a generating a single-cysteine extracellular loop. Zero or two cysteines appear in Domain II loops of cephalochordate and the most basal T-type channels (single cell coanoflagellates, placozoan, and cnidarians). Adapted from Senatore et al. (2014a).

The sequence variations, which engender sodium or calcium selectivity in NALCN channels, are consistent with prevailing evidence that suggests the HFS site and residues of the P2 helix are the principle domains governing ion selectivity in many 4X6TM channels. Alternative splicing in Domain II was also discovered for another 4x6TM channel from the same representative invertebrate species of the giant pond snail, *Lymnaea stagnalis* (Senatore et al., 2014a). But notably, this splicing in the singleton invertebrate Ca_v_3 T-Type channel, LCa_v_3, does not involve the HFS site or P2 helix. It codes for alternative exons 12a and 12b spanning the extracellular region of the pore domain, located mostly upstream of NALCN splicing. These exons code from the transmembrane helix S5, ascend as the extracellular turret to the descending pore helix P1 and terminate before the HFS site and P2 helix (Senatore et al., 2014a) (Figures 2, 7).

## Sodium ions are favored over calcium ions in T-type channels with exon 12a

**Figure 8:**
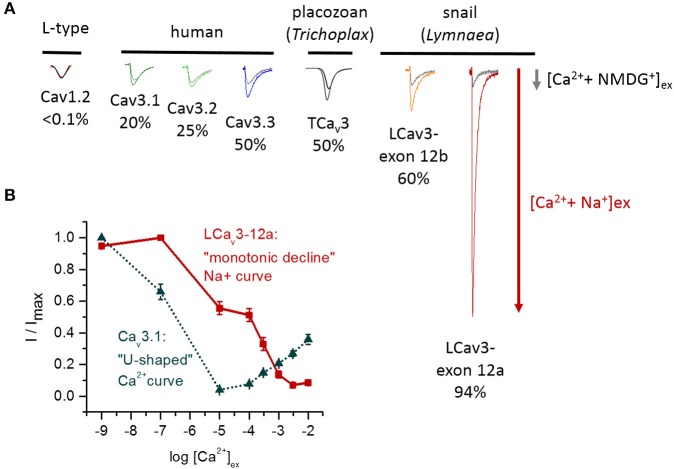
**Variable sodium selectivities in different Ca_v_3 T-type channels. (A)** The sodium current through T-type channels can be evaluated as the change of the total current when external calcium is held at physiological concentrations and external Na^+^ is added to replace membrane-impermeant NMDG^+^ ions (equimolar concentrations of Na^+^ and NMDG^+^). The sodium current ranges from 20% (Ca_v_3.1) to 94% (LCa_v_3-12a) of the total sodium and calcium current. Highly calcium-selective channels such as Ca_v_1.2 lack measureable sodium currents in the presence of calcium ions. **(B)** “Anomalous effect” experiment illustrating the competition between sodium and calcium ions that pass through the pore. The relative current (I/Imax) was measured in response to increasing external calcium concentrations (1 × 10^•9^ mM to 1 × 10^•2^ mM) in the presence of 60 mM external Na^+^ ions. The human Ca_v_3.1 channel strongly favors calcium, whereas the invertebrate T-type channel strongly favors sodium ions. The classical “U-shaped” dependence of human Ca_v_3.1 channels reflects a strong block of the sodium current (maximal at 10 μM calcium) and a rising calcium conductance in the physiological (mM) range of external calcium. The slow “monotonic decline” of the snail LCa_v_3-12a sodium current with increasing calcium concentrations reflects a weak capacity of calcium ions to block the snail T-type channel sodium current and a very weak calcium conductance in physiological range (mM) of external calcium. **(B)** Adapted from Senatore et al. (2014a).

LCa_v_3 channels expressed with exon 12a are sodium-selective T-type channels. At physiological ionic concentrations of external sodium and calcium, ~94% of the current through these channels is carried by sodium ions (Senatore et al., 2014a) (Figure 8A). When monovalent ions (Cs^+^, K^+^, Na^+^, or Li^+^) are artificially held at high concentrations (100 mM) inside the cell with physiological concentrations of calcium outside the cell, very large outward monovalent currents are generated relative to the calcium influx, with a reversal potential in these bi-ionic conditions that reflects the extremely high sodium permeability of these LCa_v_3 channels with exon 12a (Senatore et al., 2014a). The imbalance, which favors sodium ions, can be observed as competition between sodium and calcium ions for influx through LCa_v_3-12a in “anomalous effect” experiments (Senatore et al., 2014a) (Figure 8B). Typically, highly calcium selective channels pass sodium ions in the absence of calcium. However, increasing the calcium concentration to 10 μM diminishes the sodium current, reflecting the calcium block of any sodium conductance (Figure 8B). Increasing the extracellular calcium concentrations from 10 μM to the physiological (mM) levels results in the current increase, reflecting the favored and high permeability of calcium-selective channels for calcium over sodium (Figure 8B). The potent calcium block of the sodium current, and high permeability for calcium ions at physiological concentrations, creates the “U” shaped dependence of current with increasing external calcium in the presence of physiological levels of external sodium. In place of the “U” shaped anomalous effect curve, the curve for the sodium-selective LCa_v_3-12a T-type channel is shaped as a continuous monotonic decline with increasing calcium ions. This reflects a very weak capacity for calcium to block the sodium current and a relatively small calcium conductance through these channels even in the presence of physiological levels of external calcium (Senatore et al., 2014a).

## Sodium permeability from small to large levels is observed in all T-type channels

The relative sodium and calcium conductances in different expressed T-type channels can be evaluated by replacing NMDG^+^ with sodium ions in the extracellular solution that also has physiological levels of calcium. NMDG^+^ is a large impermeant monovalent ion that does not contribute an inward current, so the total inward current increase when sodium replaces NMDG^+^ indicates relative contributions of sodium and calcium conductances under these conditions (Figure 8A). A striking observation is that sodium currents contribute substantially to the total ion current in all T-type channels at physiological concentrations of calcium. This contrasts with highly calcium-selective Ca_v_1 and Ca_v_2 channels where the sodium current is immeasurably small (<1% of the current carried by sodium ions). There are three categories of relative sodium permeability in T-type channels. First, mammalian Ca_v_3.1 and Ca_v_3.2 channels are the most calcium selective ones where ~20–25% of the current is carried by sodium ions (Figure 8A). Second, mammalian Ca_v_3.3 and invertebrate T-type channels (e.g., in *Trichoplax*, *Lymnaea*) are non-selective channels that pass approximately equal mixtures of sodium and calcium ions at their physiological concentrations (Figure 8A). Third, the invertebrate T-type channel with exon 12a is unique in its resemblance to a sodium-selective channel, with a sodium selectivity >90%, approximating that of classical Na_v_1 sodium channels (Figure 8A).

## The HFS site is not the only determinant in governing ion selectivity of Ca_v_3 T-type channels

The HFS site of T-type channels has the EEDD ring, whereas highly calcium-selective Ca_v_1 and Ca_v_2 channels have the EEEE ring. The aspartate side chain is one carbon atom shorter than that of glutamate. Apparently, the shortened side chains significantly affect the HFS site ion selectivity. Indeed, the presence of the DDDD or EEEE ring in the HFS site is critical for providing sodium or calcium selectivity in homotetrameric Nav channels (Decaen et al., 2014; Finol-Urdaneta et al., 2014).

All known species with Ca_v_3 T-type and Ca_v_1 L-type channels (including the basal, single cell eukaryote, the coanoflagellate, *Sapingoeca rosetta*) bear an EEDD HFS site in their Ca_v_3 channels and EEEE HFS site in their Ca_v_1 channels. If the Ca_v_3 T-type channels evolved to provide calcium selectivity in the manner resembling Ca_v_1 channels, then there would have been at least one example of a Ca_v_1-like EEEE HFS site in T-type channels. However, such examples are not known. Engineering the Ca_v_1-like EEEE HFS site onto T-type channels generates T-type channels that are even more sodium permeable, not more calcium selective as would be expected if the HFS site were a principal determinant for ion selectivity in Ca_v_3 T-type channels (Talavera et al., 2001, 2003; Park et al., 2013).

Experiments involving T-type channels suggest that the HFS site at the ring of acidic residues between the P1 and P2 helices is not the only determinant of ion selectivity. All T-type channels possess an EEDD HFS site, but the sodium permeability ranges in T-type channels from 20% (Ca_v_3.1) to 94% (LCa_v_3-12a) (Figure 8A). Invertebrate and vertebrate Ca_v_1 and Ca_v_2 channels consistently permeate barium currents that are twice as large as calcium currents, while different T-type channels can have calcium current that is larger, smaller or equal to barium current (McRory et al., 2001; Senatore and Spafford, 2010, 2012).

The selectivity-filter region of 4x6TM channels likely resembles that in the bacterial NavAb channel, which is shorter (along the pore axis) and apparently wider than that in potassium channels (Figure 3B). Molecular dynamics simulations suggest that more than one ion can pass through the NavAb selectivity filter at the same time (Chakrabarti et al., 2013).

## Cysteine-rich extracellular turrets govern selectivity of Ca_v_3 T-type channels

Exon 12a, which generates sodium-selective T-type channels, and exon 12b, which creates a more calcium-selective T-type channel, are both located at the extracellular turret of Domain II (Figure 7). Exon 12a and exon 12b differ in their size and pattern of cysteines, while most of other residues are highly variable (Figure 7). The majority of protostome invertebrate species (within nematodes, arthropods, annelids and mollusks) possess both exons 12a and 12b, although a few species possess only exon 12a (e.g., *Brugia* nematode, *Helobdella* annelid) or only exon 12b (e.g., *Capitella* annelid) (Figure 7). Exon 12a is shorter (~41 amino acids) with a highly conserved motif containing three cysteines (CxC….C), while exon 12b is longer (~52 amino acids) and has a highly conserved motifs with five cysteines (C… CxC… CxC or CxxC… C… CxC in nematodes) (Figure 7). Our consistent findings (unpublished) are that mutating the cysteine-containing motifs of exon 12a and 12b generates highly unusual T-type channels that have a capacity to conduct multiple differing ions simultaneously and independently, as if the pore selectivity is lost.

## A conserved pattern of cysteines within extracellular turrets in 4x6TM channels

**Figure 9:**
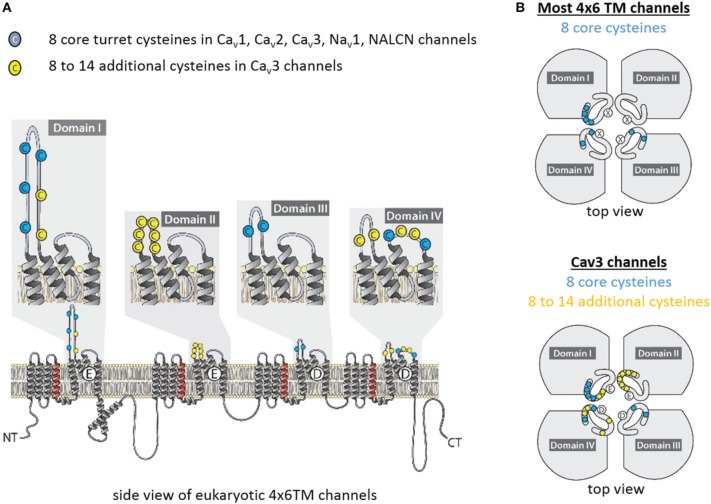
**Conserved cysteines in extracellular loops of 4x6TM channels**. T-type channels have 8–14 additional cysteines (yellow) above the core eight cysteines (blue), which are conserved amongst all 4x6TM channels (Ca_v_1, Ca_v_2, Ca_v_3, Na_v_1, Na_v_2, NALCN). **(A)** Side and **(B)** extracellular views at the cysteine distribution in the extracellular turrets.

**Figure 10:**
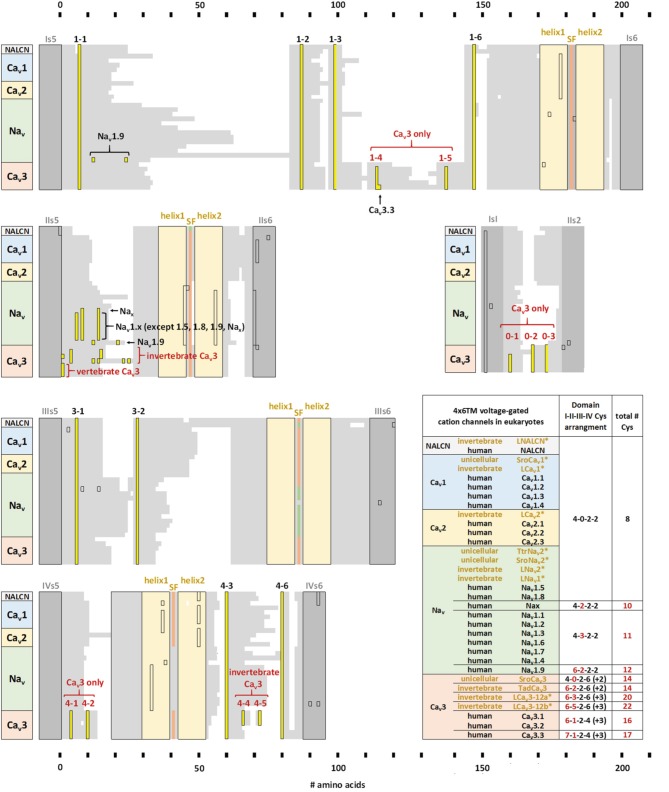
**Alignment of transmembrane helices and P-helices in 4x6TM channels illustrating location of extracellular-loop cysteines**. The pore module includes transmembrane helices S5, S6 (dark gray), and pore helices P1 and P2 (tan) on either side of the selectivity filter (SF). Conserved cysteines (yellow) are present in the variable-size extracellular loops (light gray). Eight core cysteines are located at positions 1-1, 1-2, 1-6, 3-1, 3-2, 4-3, and 4-6. Additional cysteines are found in Domain II of most vertebrate Na_v_1 channels and T-type channels. All T-type channels also have conserved cysteines in S5P loops of Domain I (1-4,1-5) and Domain IV (4-1,4-2), as well as in the extracellular loop between S1-S2 segments of Domain I. Two additional cysteines unique to invertebrate T-type channels are found in Domain IV extracellular loop PS6, C-terminal to the P2 helix (4-4, 4-5). The alignment is obtained using sequences of 21 human 4x6TM channels, five invertebrate representatives from the giant pond snail *Lymnaea stagnalis* [LNALCN, LCa_v_1, LCa_v_2, LCa_v_3-12a (-12b), LNa_v_1 and LNa_v_2], three representatives from unicellular coanoflagellate, *Salpingoeca rosetta* (SroCa_v_1, SroCa_v_3, and SroNa_v_2) and TtrNa_v_2, and the single representative from unicellular (non-animal) eukaryote, *Thecamonas trahens*.

How can the ion selectivity depend on the number and pattern of cysteines in an extracellular turret of one particular domain in invertebrate Ca_v_3 T-Type channels? There is no high resolution structure of extracellular regions in 4x6TM channels. Furthermore, the sequence and length of extracellular loops vary tremendously between individual domains in each 4x6TM channel and between different 4x6TM channels. However, an intriguing pattern of conserved cysteines is seen in aligned sequences of extracellular regions of 4x6TM channels (Figure 9). The four extracellular loops are located above (extracellularly to) the selectivity filter. Each loop links the outer helix S5 to the selectivity filter (S5P linker) and the selectivity filter to the inner helix S6 (PS6 linker). The multiple sequence alignment reveals a basal set of eight cysteines in extracellular loops that are shared in known eukaryotic 4x6TM cation channels. The set involves four, two and two cysteines in linkers DI-S5P, DIII-S5P, and DIV-PS6, respectively (Figures 9, 10). This pattern of eight conserved cysteines is found in all 4x6TM channels known to date, including the basal Na_v_2 channels from the single-cell eukaryotes, apusozoan *Thecamonas trahens* and coanoflagellate *Salpingoeca rosetta*. This pattern is also found in representative invertebrates (i.e., giant pond snail, *L. stagnalis*) and vertebrate (human) forms of NALCN, Ca_v_1, Ca_v_2, Ca_v_3, Na_v_1, and Na_v_2 channels (Figure 10).

Compared to the standard set of eight core cysteines in all 4x6TM channels, Ca_v_3 T-type channels possess 8–14 additional cysteines (Figure 10). These include seven cysteines found exclusively in Ca_v_3 channels, namely, two cysteines in DI-S5P (labeled 1-4, 1-5), two cysteines in DIV-S5P and 0, 1, 3, or 5 cysteines in DII-S5-P (Figure 9). All T-type channels also uniquely have two cysteines in Domain I S1-S2 extracellular loop of the voltage sensor domain. In addition to these T-type channel exclusive cysteines, there are two cysteines in DIV-PS6, which are specifically unique to invertebrate Ca_v_3 channels (Figure 10). Vertebrate Na_v_1 sodium channels share the features of Ca_v_3 channels in containing a variable cluster of cysteines within DII-S5P, whose number varies: zero in Na_v_1.5 and Na_v_1.8, two in Nax and Na_v_1.9, or three in Na_v_1.1, Na_v_1.2, Na_v_1.3, Na_v_1.4, Na_v_1.6, and Na_v_1.7 channels (Figure 10). The variable DII-S5P cysteine cluster in Ca_v_3 channels always contains three or five cysteines in prototstome invertebrates, two cysteines in placozoan, *Trichoplax adherens*, one cysteine in vertebrate Ca_v_3 channels, or no cysteines in more basal species such as single cell eukaryotes *S. rosetta* and cnidarians (e.g., *Nematostella vectensis*, *Hydra magnipapillata*, and *Acropora millepora*).

## Exclusive cysteines in the extracellular loops of T-type channels

The eight core cysteines in the pore module extracellular loops (S5P and PS6) shared by all 4x6TM channels seem fundamental for the ion channel integrity. Mutation of any of the four conserved cysteines in DI-S5P (1-1,1-2,1-5,1-6) of Ca_v_3.1 channels (Figure 10) creates non-functional T-type channels (Karmazinova et al., 2010). On the other hand, in human T-type Ca_v_3.1 and Ca_v_3.2 channels, mutations of exclusive cysteines in DI-S5P (1-3, 1-4) or DII-S5P of the pore module or in Domain I S1-S2 extracellular loop of the voltage sensor module, generate functional phenotypes of variable properties, consistent with modulatory roles of extracellular cysteines in channel gating and ion selectivity, (Karmazinova et al., 2010; Senatore et al., 2014a) and susceptibility to redox (Todorovic and Jevtovic-Todorovic, 2007; Karmazinova et al., 2010) modulation. Adaptation of the DII-S5P loop in invertebrates to generate sodium selective T-type channels may relate to the uniqueness of Domain II, which appears to weakly contribute to gating properties, compared to the other three pore domains.

Indeed, when either repeat Domain I, III, or IV of the Ca_v_3.1 T-type channel is replaced by the respective repeat domain from the Ca_v_1.2 L-type channel, the resulting chimeric phenotype demonstrates the high voltage of activation as observed in L-type channels (with voltage increases of 40–50 mV). In contrast, replacing Domain II of the T-type channel with Domain II from the L-type channel results in only a minor change in voltage activation, ~10 mV (Li et al., 2004, 2005). Importantly, the pore module rather than the voltage sensor module is what engenders the dramatic change of the channel voltage sensitivity upon replacement of Domain I (Li et al., 2005). This may explain why Domain II splicing with exon 12a dramatically changes the ion selectivity, but causes minor changes in other biophysical properties of the LCa_v_3-12a T-type channel (Senatore and Spafford, 2010; Senatore et al., 2014a).

We speculate that the structural changes that transform calcium selectivity into sodium selectivity in invertebrate Ca_v_3-12a channels, which are enriched with additional cysteines in the extracellular loops, are associated with inter-domain disulfide bonding. Cryo-electron microscopy of Na_v_1 (Sato et al., 2001), Ca_v_1 (Walsh et al., 2009b), and Ca_v_3 channels (Walsh et al., 2009a) provides structures whose resolution is too low to see possible disulfide bonds. However, structurally important inter-domain disulphide bonds are present in smaller channels that have been observed at higher resolutions (Brohawn et al., 2012; Miller and Long, 2012).

## Longer loops form extracellular structures above K channel pores

**Figure 11:**
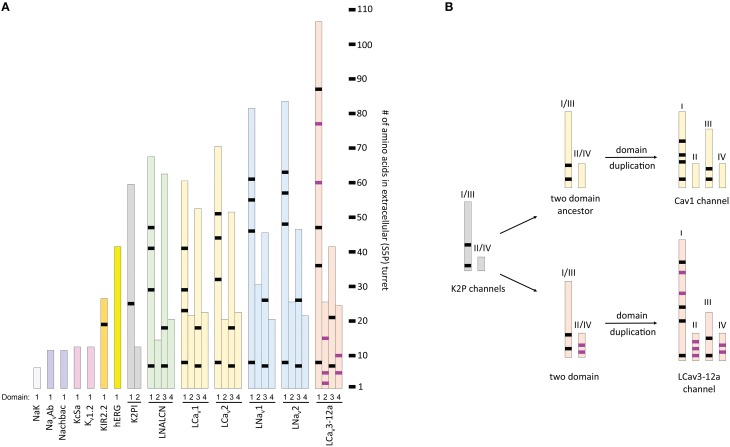
**Relative length of extracellular loops in K channels, two-pore K2P1 channel, and representative 4x6TM channels from the giant pond snail, *Lymnaea stagnalis***. Locations of shared cysteines are shown in black, while the T-type channel specific cysteines are shown in purple. **(A)** The size of extracellular turrets varies from 6 to 12 residues in bacterial K and Na channels (NaK, NavAb, NaChBac and KcsA) and Shaker-type voltage-gated K channel (Kv1.2). Longer turrets (26 residues) in the human inward rectifying potassium channel, Kir2.2, change the external landscape of the outer vestibule. Human hERG (Kv11.1) channel has a characteristic amphipathic helix in its extracellular loop (41 residues) that regulates both gating kinetics and ion selectivity. Domain D1 turret in of the K2P channel is much longer (60 residues) than domain D2 turret (12 residues). The long D1-D1 turrets form a helical cap above the pore, bridged by a disulphide bond (Brohawn et al., 2012; Miller and Long, 2012). **(B)** The long-short-long-short configuration of loops in repeat domains DI-DII-DIII-DIV of 4x6TM channels resembles that in the two domain K2P channel, after undergoing a domain duplication (see inset). Evidence for a kinship between DI-DIII and DII-DIV loops pairs is the conservation of the six core S5P cysteines within all 4x6TM channels in the longer DI-S5P:DIIIS5P pair. T-type channels differ from other 4x6TM channels by having a longer DI loops with additional cysteines and presence of T-type channel specific cysteines in the DII-S5P: DIV-S5P pair.

Long extracellular loops may form extracellular appendages above the pore. The smallest S5P turrets are short, 6–12 residues (Figure 11). Short S5P turrets are associated with bacterial channels (e.g., NaK, KcsA, and NavAb) and eukaryotic voltage-gated potassium channels (e.g., *Shaker* and Kv1.2) (Figure 11). Eukaryotic inward rectifying potassium channels like Kir2.2 have longer S5P loops, more than twice as long as those in the bacterial NavAb channel (Figure 11). The longer loops alter the extracellular landscape enough to reduce binding of classical potassium channel drugs, like tetraethylammonium (TEA) (Tao et al., 2009). S5P loops, which are ~3.5 times longer than in NavAb, are present in Kv11 hERG K channels (Figure 11), and these contain a unique amphipathic helix(Torres et al., 2003). The presumably flexible amphipathic helices are suggested to contribute to the pore collapse associated with rapid C-type inactivation, which is characteristic for hERG channels (Torres et al., 2003).

Some S5P mutations in the hERG channels can dramatically alter the ion selectivity, and make the channels highly permeant to sodium ions, suggesting that turret residues may affect the selectivity filter like in T-Type channels (Liu et al., 2002).

**Figure 12:**
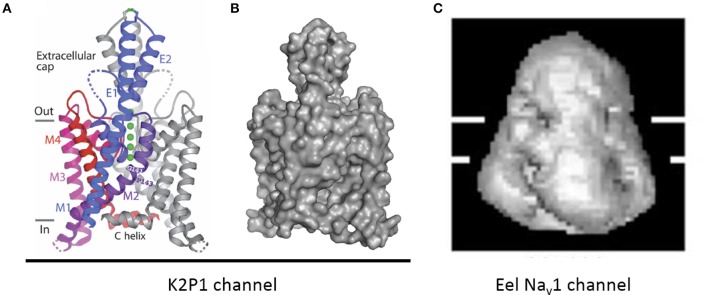
**An extracellular appendage above the selectivity filter in K2P1channel. (A)** The X-ray structure (PDB code 3UKM) shows an extracellular cap extending 35 Å above the plane membrane, with inter-subunit disulfide bond at the apex. The cap limits drug access to the pore. Ions move through side portals, which serve as a pre-filter. Reproduced with permission from Miller and Long (2012). **(B)** Surface image of the of the K2P1 channels generated with PyMoL from the PDB coordinates 3UKM. **(C)** Single particle cryo-electron microscopy image of the electric eel Na_v_1 channel at 19 Å resolution reproduced with permission from Sato et al. (2001). Horizontal lines demarcate the boundaries of trans-membrane helices. The large extracellular bell-shape cap above the membrane, which is reminiscent of the extracellular cap in K2P channels, can be formed by pairing of long extracellular turrets. Note different scale of images **(B,C)**.

The two-pore 2x2TM K2P leak channels have two domains, each domain comprising four transmembrane helices. The M1-P1 extracellular linker in the K2P channel, likely the longest one among potassium channels, is ~5 times longer than the S5P linker in NavAb (Figure 11). The alpha-helix in the M1-P1 linker is capped by a cysteine that forms a disulfide bond with a cysteine capping the second-domain M3-P2 helix (Figure 12). The two salt-bridged helices create an extracellular appendage that forms a “carafe plug” above the pore selectivity filter (Miller and Long, 2012).

## Possible similarities between extracellular appendages in K2P and 4x6TM channels

The S5P extracellular loops of 4x6TM channels may fold in a way resembling the extracellular appendage in the K2P channels. Domain I and domain III S5P loops in 4x6TM channels are the longest ones and, like the long extracellular turrets of the K2P channels, they may be diametrically opposed above the outer pore (Figure 12A). DI-S5P and DIII-S5P loops range in size and in the T-type channels they have approximately twice as many amino acids as K2P helical turrets (Figure 11). All 4x6TM channels share six core cysteines in extracellular loops DI-S5P and DIII-S5P, and it is tempting to speculate that the loops are bridged through their conserved cysteines.

The smallest pair of loops is DII-S5P and DIV-S5P (Figure 11). A majority of the T-type channel specific cysteines are located in these loops of approximately equal size. We speculate that the extracellular turrets in DII and DIV may diametrically oppose each other, contributing to a structural appendage that is unique to the T-type channels. Perhaps, this appendage is contributing to variable sodium selectivity amongst different invertebrate and vertebrate T-type channels.

Extracellular loop pairing DI-DIII and DII-DIV in 4x6TM channels is consistent with the evolutionary kinship of these domains. These pairs formed 2x6TM channel intermediates in a manner resembling the K2P channel or Transient Receptor Potential channel dimer, prior to a second duplication event, which generated a four-domain 4x6TM channel (Figure 11, inset). A longer loop with conserved cysteines might have evolved within the first of two domains in the two domain intermediate of 4x6TM channels (Figure 11, inset). The second, shorter set of domains may have become infused with extra cysteines in a possible T-type channel ancestor (Figure 11, inset).

## Ion selectivity in 4x6TM channels with cysteine-rich extracellular turrets

Structure of K2P channels might suggest how extended turrets fold in 4x6TM channels. The extracellular cap structure in K2P channels bridged by disulphide bonds in extended turrets limits ion access to cation-attractive side portals of variable sequence that serve as a variable pre-filter for cations funneling to the pore selectivity filter below (Miller and Long, 2012) (Figure 12A). The C-terminal end of the second extracellular helix of the K2P turret approaches the pore selectivity filter just above and to the side (Miller and Long, 2012). The closeness of the turret to the selectivity filter suggests that ion selectivity could be regulated both by the pre-filtering of ions involving charged residues lining the conduit in side portals within the extracellular scaffold and by influences of negatively charged turret residues positioned next to the pore selectivity filter.

The highest resolution image of a 4x6TM channel is obtained for the electric eel Na_v_1 channel at 19 Å with single particle cryo-electron microscopy (Sato et al., 2001) (Figure 12C). A large extracellular appendage of the bell-shaped Na_v_1 channel above the membrane (Figure 12C) that resembles the extracellular cap of the K2P channels (Figure 12B) may be formed by extracellular S5P turrets in Domains I and III. We speculate that the ion selectivities within T-type channels may be affected by exons in Domains I and III as well as the specific cysteine arrangement of extracellular loops in Domains II and IV.

## Cysteine-rich extracellular turrets may affect access of drugs and toxins to the outer pore

Venomous animals synthesize cocktails of specific toxins against most classes of ion channels and non-channel targets. Examples of calcium channel targeting toxins include spider agatoxins that block P/Q-type channels, snail ω-conotoxins that block N-type channels, spider toxin SNX-482 that blocks R-type channels, and snake venom calciseptine that blocks L-type channels (Catterall et al., 2007).

Peptide toxins with reported effects on T-type channels, such as ProTx-I and ProTx-II (Bladen et al., 2014), and kurtoxin (Sidach and Mintz, 2002) are also effective on other calcium channel types, and most notably Na_v_1 sodium channels.

The extended turrets of the Kir2.2 channels (Tao et al., 2009) and K2P channels (Miller and Long, 2012) form a cap that alters drug and toxin access to the outer pore. Both Kir and K2P channels are resistant to external block by potassium channel drugs like TEA.

Na_v_1.x channels have big extracellular loops, which do not prevent TTX or peptide toxins to target the outer pore. For example, mu-conotoxin GIIIA binds to the outer carboxylates in the Na_v_1.4 channel (see Korkosh et al., 2014 and references therein), which have big extracellular loops. Apparently, if the loops are disulfide-bridged, the appendage should have openings wide enough to let the toxins through to the outer pore.

Scorpion toxins within the delta-KTx family specifically target the hERG extracellular loops. These toxins are distinct from charybdotoxin (alpha-KTx family of toxins), which plugs the ERG channel pore (Vandenberg et al., 2012). T-type channels may resemble the Kir2.2 and K2P channels, because no naturally-occurring peptide toxins have been isolated that specifically target the pore module of T-type channels.

High throughput screening methods have revealed many T-Type channel specific blockers (Giordanetto et al., 2011), including Z944 (Tringham et al., 2012) and TTA-P2 (Dreyfus et al., 2010). High resolution structures obtained with direct detection camera and single nanoparticle cryo-electron microscopy (Liao et al., 2014) may help clarify the structural peculiarities of extracellular loops that underlie the diversity within different T-type channels, as well as between T-type channels and other 4x6TM channels. Knowledge of the extracellular structures above T-type channels may also assist in future drug design.

## Conclusions

Invertebrates have evolved to generate sodium and calcium selectivity within the family of 4x6TM channels. Invertebrate species normally have a singleton gene for NALCN, but three genes for calcium channels (Ca_v_1, Ca_v_2, and Ca_v_3) and two sodium channel genes (Na_v_2, Na_v_1). NALCN resembles a calcium channel in basal metazoans, a sodium channel in animal groups such as vertebrates, and bears both dual sodium and calcium selective pores in most invertebrates (Senatore et al., 2013). The replacement of sodium and calcium selectivity in invertebrate NALCN channels by splicing at HFS site confirms this site with a lysine in the second or third domain as a key determinant of the sodium selectivity in 4x6TM channels. This splicing affects the same HFS site where artificial swapping of selectivity filter residues between mammalian Ca_v_1.2 and Na_v_1.2 channels has been demonstrated to change the ion selectivity (Heinemann et al., 1992; Schlief et al., 1996).

Invertebrate Ca_v_3 T-type channels also generate alternative sodium or calcium selectivity due to the extracellular S5P loop in Domain II, which is located above the HFS site and P2 helix (Senatore et al., 2014a). 4x6TM channels have cysteines in the extracellular loops of the diametrically opposing domains. In K2P channels, such cysteines form a disulfide bond to stabilize an extracellular appendage above the selectivity filter that serves as a pre-filter for permeating ions and as a shield that limits drug and toxin access to the selectivity filter. Consistent with this idea, cryo-electron microscopy of the eel Na_v_1 channel shows a dome-shaped extracellular appendage. It is tempting to speculate that such extracellular appendages exist above the pores of eukaryotic 4X6TM channels, and that T-type channels have a unique extracellular appendage that contributes to alternative sodium or calcium selectivity. Various cysteine-rich loops in different T-type channels appear to provide different ion selectivity properties and may regulate drug access. A possible avenue for future drug design is targeting the variable loop sequences. There is more variability between different T-type channels in the extracellular loop regions than in the selectivity-filter and inner pore regions, so targeting the loop sequences could facilitate design of drugs. In the future, single nanoparticle cryo-electron microscopy and advanced direct detection cameras may provide greater insights into the nature of the variable extracellular appendages above 4x6TM channels.

### Conflict of interest statement

The authors declare that the research was conducted in the absence of any commercial or financial relationships that could be construed as a potential conflict of interest.

## Abbreviations

1x6TM: a subunit of six transmembrane helices found in voltage-gated K channels, and prokaryotic voltage-gated sodium channels;
4x6TM: four repeat domains, each domain having six transmembrane helices found in voltage-gated sodium channels, calcium channels, and NALCN;
S1–S4: four transmembrane helices that form the voltage sensor domain;
P-loop: the extracellular membrane-reentering loop between S5 and S6. It includes S5P, selectivity filter, and PS6;
S5P: the extracellular loop between transmembrane helix S5 and the pore helix P1;
PS6: the extracellular loop between the pore selectivity filter and transmembrane helix S6;
P1 and P2 helices: the pore helices flanking the selectivity filter residues;
DI, DII, DIII, DIV: Four repeat domains in 4x6TM channels;
HFS: High Field Strength site within the selectivity filter;
Na_v_1: sodium-selective, voltage-gated sodium channels, that are limited to extant animals with nervous systems;
Na_v_2: the calcium-selective, voltage-gated sodium channel gene, first cloned and expressed in cockroach, *Blatella* BSC1, fruit fly, *Drosophila* as DSC1, and *Nematostella* Na_v_2.1-2.4 and notably absent in vertebrates. It is the most basal sodium channel, appearing in single cell eukaryotes and should not be confused with Nav2, neuron navigator 2, found in vertebrates, only;
Ca_v_1 (L-type) and Ca_v_2 (Non-L-type): calcium-selective voltage-gated calcium channels;
Ca_v_3 (T-type): voltage gated channel structurally intermediate between calcium (Ca_v_1/Ca_v_2) and sodium (Na_v_2/Na_v_1) channels. Ca_v_3 can be calcium-selective (vertebrates) or highly sodium-selective (exon 12a isoform in invertebrates);
NALCN: Sodium (Na) Leak ChaNnel, an orphan channel usually a single gene in most species. It is more similar to yeast calcium transporter CCh1P than to 4x6TM channels. Mammalian NALCN is also known as “Voltage-Independent Sodium Channel 2.1” (Na_VI_2.1) and “Voltage gated channel-like protein 1 (VGCNL1). In fruit fly *Drosophila melanogaster*, it has been described as “Unknown- or U-type (Dmα1U)” channel, or by its mutant phenotype “narrow abdomen” or “na.” The two genes in *Caenorhabditis elegans*, are known as nematode cation channel 1 and 2 (nca-1/nca-2);
KcsA: a prokaryotic pH-gated potassium channel from the soil bacteria *Streptomyces lividans*;
NavAb: the 1x6TM prokaryotic voltage-gated sodium channel isolated from *Arcobacter butzleri*. NavAb ia a close homolog to NaChBac from *Bacillus halodurans*;
K2P: two pore potassium voltage-independent channels, Include six families: TWIK, TREK, TALK, TASK, THIK, TRESK;
hERG: human voltage-gated potassium channel Kv11. EAG stands for ether-a-go-go.
